# Feasibility of 4D-flow CMR for haemodynamic characterization in hypertrophic cardiomyopathy after septal myectomy with and without anterior mitral valve leaflet extension

**DOI:** 10.1093/icvts/ivae210

**Published:** 2024-12-16

**Authors:** Sulayman el Mathari, Pim van Ooij, Renske Merton, Eric Schrauben, Luuk Hopman, Aart Nederveen, Marco Götte, Jolanda Kluin

**Affiliations:** Department of Cardiothoracic Surgery, Amsterdam University Medical Center, Amsterdam, The Netherlands; Department of Cardiothoracic Surgery, Erasmus University Medical Center, Rotterdam, The Netherlands; Department of Radiology and Nuclear Medicine, Amsterdam University Medical Center, Amsterdam, The Netherlands; Department of Radiology and Nuclear Medicine, Amsterdam University Medical Center, Amsterdam, The Netherlands; Department of Radiology and Nuclear Medicine, Amsterdam University Medical Center, Amsterdam, The Netherlands; Department of Cardiology, Amsterdam University Medical Center, Amsterdam, The Netherlands; Department of Radiology and Nuclear Medicine, Amsterdam University Medical Center, Amsterdam, The Netherlands; Department of Cardiology, Amsterdam University Medical Center, Amsterdam, The Netherlands; Department of Cardiothoracic Surgery, Erasmus University Medical Center, Rotterdam, The Netherlands

**Keywords:** 4D-flow CMR, obstructive hypertrophic cardiomyopathy, septal myectomy, mitral valve repair, anterior mitral valve leaflet extension, haemodynamic differences

## Abstract

**OBJECTIVES:**

The common surgical treatment in patients with obstructive hypertrophic cardiomyopathy is septal myectomy. This involves resection of a segment of the myocardial septum and can be performed with and without concomitant anterior mitral valve leaflet extension (AMVLE). While both approaches have satisfying clinical outcomes, there is a lack of data regarding the added value of concomitant AMVLE. In particular, their impact on postoperative haemodynamics remains unexplored. Therefore, we conducted a study to assess the feasibility of utilizing four-dimensional-flow cardiac magnetic resonance imaging (4D-flow cardiac magnetic resonance imaging (CMR)) to investigate postoperative haemodynamic differences among both surgical approaches.

**METHODS:**

In this feasibility study, nine subjects underwent 4D-flow CMR evaluation, including three patients who underwent isolated myectomy, three patients with myectomy + AMVLE and three healthy controls. Primary end-points were aortic wall shear stress, left ventricular outflow tract (LVOT) peak velocity and peak kinetic energy in the LVOT and ascending aorta.

**RESULTS:**

Results showed that patients who underwent myectomy with concomitant AMVLE exhibited (i) lower aortic wall shear stress (−21.2%), (ii) lower LVOT peak velocity (−6.3%), (iii) higher kinetic energy in the LVOT (+10.8%) and (iv) lower kinetic energy in the ascending aorta (−28.8%) compared to patients who underwent isolated myectomy.

**CONCLUSIONS:**

Patients undergoing additional AMVLE exhibited a better trend towards the haemodynamic reference values from healthy controls compared to patients undergoing isolated myectomy. Our findings underscore the feasibility of 4D-flow CMR to assess postoperative haemodynamic differences in hypertrophic cardiomyopathy patients undergoing different surgical approaches. This highlights the potential of 4D-flow CMR to compare surgical strategies based on postoperative haemodynamics.

**Clinical registration number:**

Dutch National Medical Ethics Committee, registration number 2022.0078

## Graphical Abstract

**Figure:**
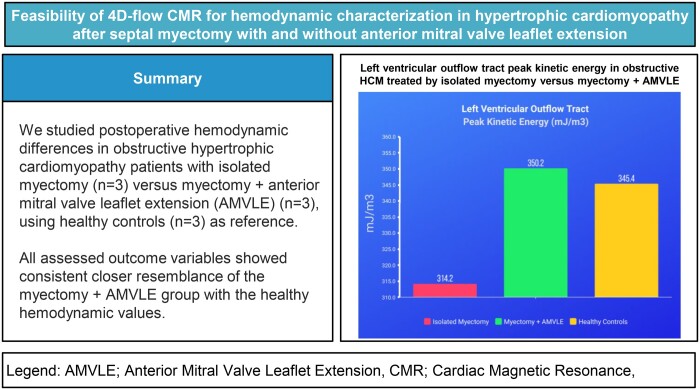

## INTRODUCTION 

Hypertrophic cardiomyopathy (HCM) is characterized primarily by thickening of the left ventricular (LV) wall, with thickness ≥15 ml in one or more segments of the LV myocardium [1]. A frequently diagnosed variant within the spectrum of HCM is obstructive HCM, distinguished by the presence of an obstruction in the left ventricular outflow tract (LVOT) [2]. This obstructive phenotype is attributable to the phenomenon of systolic anterior motion (SAM) of the mitral valve (MV), a paradoxical event wherein the anterior MV leaflet is drawn towards the LVOT during the systolic phase [3].

The common surgical procedure in symptomatic patients with HCM is septal myectomy [3]. This surgical intervention involves resection of a segment of the myocardial septum. In cases involving SAM, septal myectomy can be done as a standalone procedure or in combination with MV repair (MVR). MVR typically involves anterior mitral valve leaflet extension (AMVLE) to enlarge the leaflet tissue surface, thereby moving it away from the LVOT in the systolic phase to enhance the LVOT openness [4, 5]. Multiple studies have investigated potential differences in clinical outcomes between septal myectomy with and without concomitant AMVLE, and both techniques have proven to be clinically safe and effective [6–9]. Nevertheless, despite several decades of experience with both these procedures [10], there remains limited knowledge regarding their impact on blood flow dynamics in the LVOT and in the ascending aorta [11, 12].

Over the past decades, cardiac magnetic resonance imaging (CMR) became the standard for assessing HCM patients [13]. More recently, innovative CMR techniques have emerged, specifically the introduction of time-resolved three-dimensional (3D) phase-contrast CMR, incorporating three-directional velocity encoding, referred to as four-dimensional (4D)-flow CMR. This technology enables quantification of blood volumetric velocity vector fields within the heart and major blood vessels [14]. These vector fields can calculate haemodynamic parameters, such as wall shear stress (WSS), peak velocity and kinetic energy (KE), which are altered in disturbed flow [15, 16]. This technique may help to elucidate postoperative haemodynamic differences between the isolated myectomy and myectomy with concomitant AMVLE approaches in patients with HCM.

Therefore, we employed a 4D-flow CMR study to assess the feasibility of examining haemodynamic differences among HCM patients who underwent surgical myectomy with and without AMVLE. For this purpose, we assessed aortic WSS, variances in LVOT peak velocity, and KE in the LVOT and ascending aorta. These measurements were subsequently compared to healthy controls. Our hypothesis posited that 4D-flow CMR is a valuable technique to assess postoperative haemodynamic differences between septal myectomy with and without AMVLE. Furthermore, we postulate that myectomy in combination with AMVLE might result in haemodynamics similar to those observed in healthy individuals, due to the potential enhanced openness of the LVOT.

## METHODS

### Study population

This single-centre feasibility study with a case–cohort design involves obstructive HCM patients who underwent (i) isolated surgical myectomy or (ii) myectomy with concomitant AMVLE at the Department of Cardiothoracic Surgery, Amsterdam University Medical Center (AmsterdamUMC). Concomitant AMVLE was performed when there was significant SAM of the MV, along with expected residual mitral regurgitation or suboptimal LVOT haemodynamics (both based on expert opinion) that could not be adequately addressed by septal myectomy alone. At the moment of inclusion, participants already underwent surgery between January 2019 and May 2022 (retrospective collection), and subsequently, all patients underwent prospective 4D-flow CMR assessment in November 2022. Exclusion criteria were patients with a history of cardiac surgery and major comorbidities. A control series comprising healthy volunteers was employed to establish reference data accurately representing the healthy state. Baseline and perioperative details of all included patients are included in Supplementary Material, Table S1.

### Ethics statement

The institutional ethics committee of the Amsterdam UMC, The Netherlands, approved this study (number 2022.0078, date of approval 5-05-2022), and written patient consent was obtained to allow for pseudo-anonymous data collection and analysis.

### Outcome measures

Primary outcome measures were (i) aortic WSS (Pa), (ii) peak velocity (m/s) within the LVOT and (iii) KE (mJ/m^3^) [17] in the LVOT and ascending aorta. All primary outcomes were measured in the peak systolic phase. These haemodynamic parameters are clinically significant, as higher aortic WSS may indicate abnormal flow patterns leading to endothelial dysfunction and increased cardiovascular risk, while too low WSS can suggest stagnation and potential thrombus formation. Peak velocity serves as a critical marker for the severity of outflow obstruction; higher velocities are associated with worse symptoms and functional capacity. Lastly, KE reflects the dynamic energy of blood flow, where higher values indicate obstruction, potentially increasing myocardial oxygen demand and risk of arrhythmias, whereas lower KE suggests inadequate forward flow, risking compromised perfusion. By utilizing 4D-flow CMR to monitor these parameters, clinicians can gain valuable insights into a patient’s haemodynamic status and improve long-term outcomes in those with obstructive HCM.

Secondary outcomes were valvular flow across the aortic valve (AV) and MV, LVOT mean and maximum pressure gradient (mmHg) calculated by the simplified Bernoulli formula [18], and LVOT longitudinal strain. Key patient characteristics were evaluated by assessing standard CMR parameters, involving LV ejection fraction (LVEF), LV stroke volume (LVSV), LV end-systolic volume (LVESV), LV end-diastolic volume (LVEDV), and myocardial mass. Notably, these parameters were not obtained in the CMR protocol for the healthy controls in this study, as these were retrospectively collected. Therefore, to establish reference values for the healthy group, guidelines from the European Society of Cardiology [19] were utilized. Additionally, as part of the standard cardiac parameters, assessments were made for LVOT diameter, septal thickness, and free wall thickness.

### Cardiac magnetic resonance imaging

Scans were acquired using a 3.0 Tesla CMR system in combination with a 16-channel torso and eight-channel posterior coil (Ingenia Philips, Best, The Netherlands). Respiratory and electrocardiographic-gated 4D-flow CMR was performed to measure time-resolved 3D blood flow velocities with full volumetric coverage of the heart, with prospective undersampling in multiple directions (PROUD) acceleration as previously described [20, 21]. Pulse sequence acquisition parameters were as follows: repetition time 3.94–4.18 ms; echo time 1.98–2.18 ms; flip angle 8°; in-plane resolution 2.4 × 2.4 mm; slice thickness 2.5 mm. Data were reconstructed to 24 cardiac time frames, resulting in temporal resolution of 35–52 ms per cardiac phase. Furthermore, steady-state free precession (SSFP) cine images were acquired in short-axis, two-chamber, three-chamber and four-chamber views. Acquisition parameters for SSFP were: spatial resolution 1.5–1.8 × 1.5–1.8 × 6–8 mm^3^; temporal resolution, 35–45 ms; echo time/repetition time/flip angle, 1.1 ms/39 ms/80°.

### Data analysis

**Figure 1:**
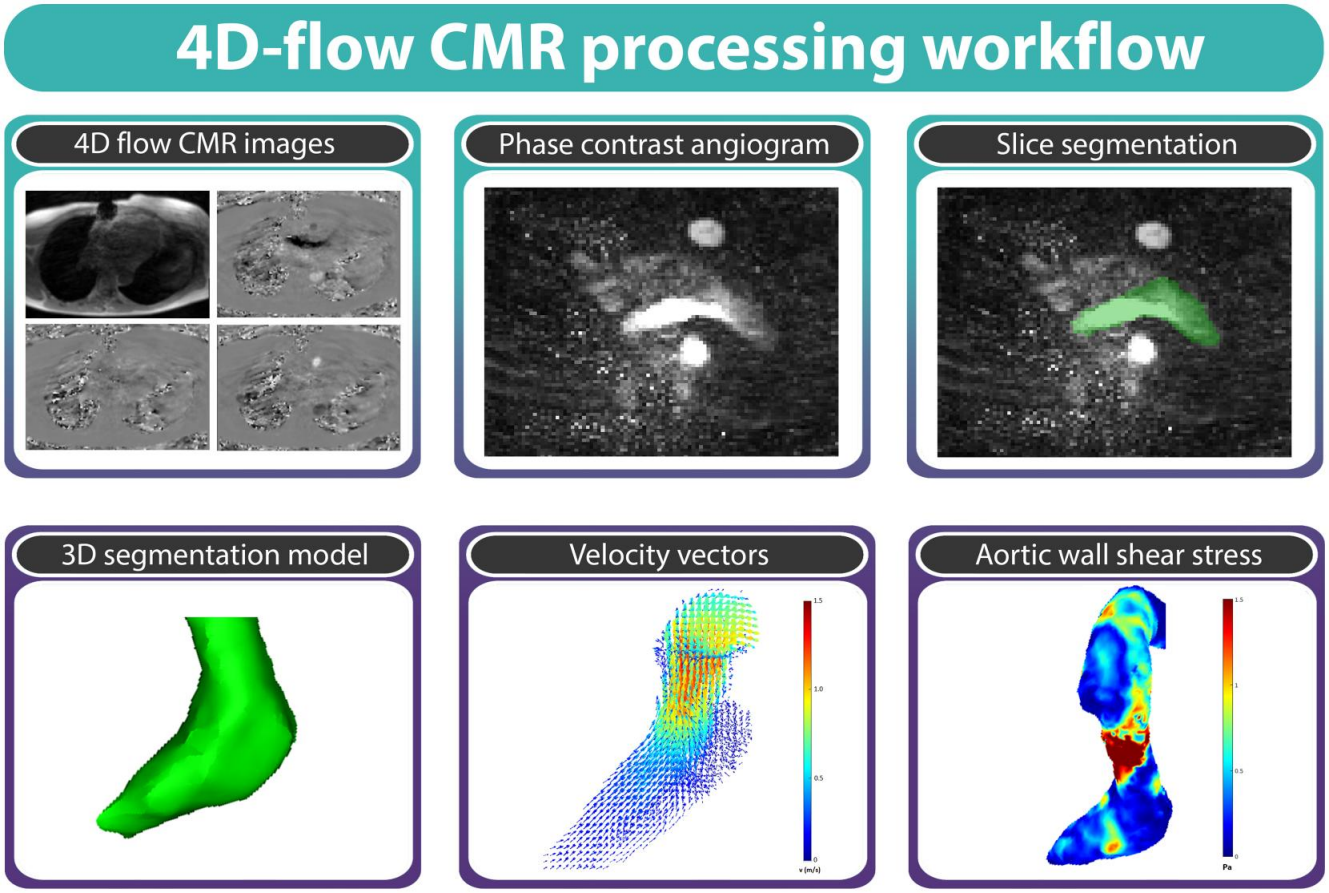
The 4D-flow cardiac magnetic resonance imaging (CMR) processing workflow encompasses the acquisition of 4D-flow CMR images and subsequent slice segmentation on phase-contrast angiogram images, yielding a three-dimensional (3D) segmentation of the left ventricle (LV) and ascending aorta. The ventricular peak systole time frame is identified through the determination of maximum averaged velocity within the segmentation, accompanied by velocity vectors and aortic wall shear stress analysis.

Preprocessing of 4D-flow CMR data involved corrections for background phase offsets and velocity aliasing. To create 3D phase-contrast angiograms, phase-contrast magnitude images were multiplied with absolute velocity images and then averaged over time. The LV, LVOT and ascending aorta were manually segmented from these images using Mimics (Materialise, Leuven, Belgium). The peak systolic phase was identified as the cardiac time frame with the highest averaged velocity in the LV, within the first half of the cardiac cycle. Fig. 1 provides an overview of this processing workflow. Additionally, we determined aortic WSS, LVOT peak velocity and KE in the LVOT and ascending aorta [22].

**Figure 2:**
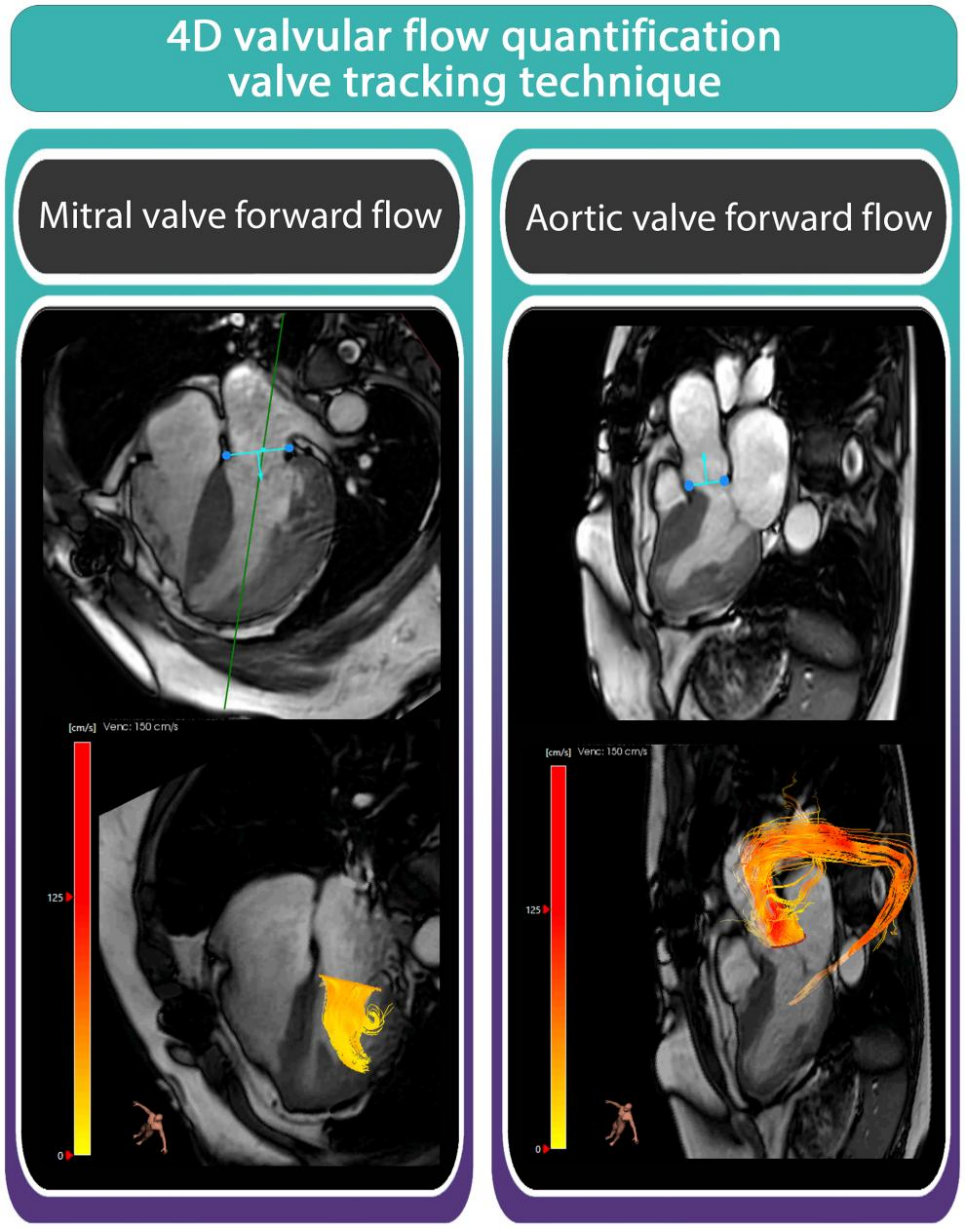
The four-dimensional valvular flow quantification involves valve tracking, a method employed for measuring mitral valve and aortic valve forward flow.

Valvular flow was quantified, as secondary outcome measure, by means of valve tracking using dedicated software (Caas MR Solutions version 5.2.1—4D-flow; Pie Medical Imaging) using two-dimensional cine SSFP images. The AV and MV were tracked on two-chamber, three-chamber and four-chamber cine views, wherein the annulus location was manually identified. Subsequently, automated tracking of valve motion was executed throughout the cardiac cycle. Following this, a time-resolved 3D plane was reconstructed and aligned with the 4D-flow CMR data. Subsequently, forward and backward blood flow were quantified in millilitres per heartbeat over the AV (Video 1) and MV (Video 2) (Fig. 2) [23].

The other secondary outcome measures (LVOT mean/max pressure gradient, LVOT longitudinal strain and LVOT V_max_) were assessed using cine SSFP images and Circle Cardiovascular Imaging software (Calgary, Alberta, Canada). The same SSFP images and analysis software were employed for quantitative measurement of the standard cardiac parameters.

### Statistical analysis

The study outcomes were presented in the form of mean values accompanied by corresponding standard deviations (SD) and 95% confidence intervals (CI). Due to the limited sample size in this feasibility study, we present our data descriptively without conducting statistical tests. This descriptive approach is sufficient for the purposes of our study, and it enhances the interpretability and accessibility of the outcomes without the risk of overstating the clinical significance of our findings.

## RESULTS

### Patient characteristics

Six study patients were included, consisting of three individuals who underwent isolated myectomy (mean age: 58.6 ± 9 years, two females) and three patients who underwent myectomy with concomitant AMVLE (mean age: 67 ± 5 years, two females). Postoperative mean follow-up time in the isolated myectomy group was 0.7 ± 0.2 years, while in the myectomy + AMVLE group, it was 2.7 ± 0.8 years. Additionally, three healthy controls were included with a mean age of 29.3 ± 2 years (two females).

Table 1 summarizes the key patient characteristics of the study population. LV volumetric parameters in the isolated myectomy group (LVESV 55.2 ± 13 ml, LVEDV 150 ± 33 ml) were higher compared to the myectomy + AMVLE group (LVESV 45.7 ± 7 ml, LVEDV 118.5 ± 8 ml). LV septal thickness and LVEF were comparable between both groups. None of the subjects suffered postoperatively from clinical complaints, residual MV regurgitation or SAM.

**Table 1:** Key patient characteristics

Patient characteristics	Isolated myectomy, *n* = 3	Myectomy + AMVLE, *n* = 3	Healthy controls, *n* = 3
Age (years ± SD)	58.6 ± 9	69 ± 5	29.3 ± 2
Female gender, *n* (%)	2 (66.7%)	2 (66.7%)	2 (66.7%)
Follow-up time since surgery (years ± SD)	0.7 ± 0.2	2.7 ± 0.8	–
Left ventricular ejection fraction (%)	63.3 ± 2	61.6 ± 4	55–70^a^
Left ventricular stroke volume (ml)	94.8 ± 21	72.8 ± 4	60–100^a^
Left ventricular end-systolic volume (ml)	55.2 ± 13	45.7 ± 7	50–100^a^
Left ventricular end-diastolic volume (ml)	150 ± 33	118.5 ± 8	46–150^a^
Left ventricular myocardial mass (g)	192.4 ± 99	122.7 ± 14	67–224^a^
LVOT diameter (mm)	18.8 ± 4.1	15.9 ± 0.9	19.6 ± 1.1
LVOT basal septal thickness (mm)	9.9 ± 1.7	10.4 ± 1.5	7.1 ± 1.5
LVOT free wall thickness (mm)	8.7 ± 1.6	7.8 ± 0.5	7.3 ± 0.5

Data are presented as means ± standard deviation and *n* (%).

a Values for healthy controls based on reference values from the European Society of Cardiology guidelines.

AMVLE: anterior mitral valve leaflet extension; LVOT: left ventricular outflow tract.

### Primary outcome measures assessed by 4D-flow cardiac magnetic resonance imaging

Aortic WSS was 0.73 ± 0.1 Pa (95% CI: [0.54, 0.92]) in the isolated myectomy group, whereas it was 0.59 ± 0.04 Pa (95% CI: [0.53, 0.65]) (−21.2%) in the myectomy + AMVLE group. Aortic WSS in healthy controls was 0.47 ± 0.01 Pa (95% CI: [0.45, 0.49]). The mean LVOT peak velocity in patients who underwent isolated myectomy was 1.63 ± 0.3 m/s (95% CI: [1.36, 1.90]), compared to 1.53 ± 0.02 m/s (95% CI: [1.51, 1.55]) (−6.3%) in those who underwent myectomy + AMVLE. Healthy controls exhibited a mean LVOT peak velocity of 1.25 ± 0 m/s.

The isolated myectomy group demonstrated a mean peak KE of 314.2 ± 62 mJ/m^3^ (95% CI: [250.2, 378.2]) in the LVOT, while the myectomy with concomitant AMVLE group recorded 350.2 ± 106 mJ/m^3^ (95% CI: [244.2, 456.2]) (+10.8%). Healthy controls showed a mean LVOT peak KE of 345.4 ± 33 mJ/m^3^ (95% CI: [317.6, 373.2]). In the ascending aorta, the isolated myectomy group exhibited a mean peak KE of 480.8 ± 68 mJ/m^3^ (95% CI: [419.2, 542.4]), contrasting with 359.9 ± 51 mJ/m^3^ (95% CI: [308.9, 410.9]) (−28.8%) in the myectomy with concomitant AMVLE group. The healthy controls resembled the latter group the most, with a mean aortic peak KE of 345.4 ± 33 mJ/m^3^ (95% CI: [317.6, 373.2]).

Notably, across all assessed primary outcome values, the group that underwent myectomy with concomitant AMVLE most closely resembled the measured values in the healthy controls (Table 2).

**Table 2:** Summary of primary outcome measures assessed by 4D-flow CMR

Primary outcomes	Isolated myectomy, *n* = 3	Myectomy + AMVLE, *n* = 3	Healthy controls, *n* = 3
LVOT peak kinetic energy (mJ/m^3^)	314.2 ± 62	350.2 ± 106	345.4 ± 33
LVOT peak velocity (m/s)	1.63 ± 0.3	1.53 ± 0.02	1.25 ± 0
Aortic peak kinetic energy (mJ/m^3^)	480.8 ± 68	359.9 ± 51	345.4 ± 33
Aortic wall shear stress (Pa)	0.73 ± 0.1	0.59 ± 0.04	0.47 ± 0.01

Data are presented as means ± standard deviation.

AMVLE: anterior mitral valve leaflet extension; LVOT: left ventricular outflow tract.

### Secondary outcome measures assessed by standard cardiac magnetic resonance imaging

In the myectomy + AMVLE group, AV forward flow was 90 ± 9 ml, and MV forward flow was 74 ± 12 ml versus 63 ± 6 ml and 84 ± 19 ml, respectively, in isolated myectomy patients. Mean and maximum pressure gradient in the isolated myectomy group were 2 ± 1.6 mmHg and 11 ± 7.9 mmHg, respectively, while in the myectomy + AMVLE group, these values were 1.6 ± 0.5 mmHg (−22.2%) and 8.6 ± 3.1 mmHg (−24.5%). LVOT longitudinal strain in the isolated myectomy patients is −10.4 ± 3.7% compared to −12.8 ± 1.7% (+20.7%). Remarkably, here again, across all assessed outcome measures, the myectomy + AMVLE group resembles the most with the measurement values in the healthy controls (Table 3).

**Table 3:** Summary of secondary outcome measures assessed by standard CMR

Baseline characteristics and results	Isolated myectomy, *n* = 3	Myectomy + AMVLE, *n* = 3	Healthy controls, *n* = 3
Valvular flow			
Mitral valve forward flow (ml)	84 ± 19	74 ± 12	83 ± 13
Aortic valve forward flow (ml)	63 ± 6	90 ± 9	86 ± 21
LVOT mean pressure gradient (mmHg)	2 ± 1.6	1.6 ± 0.5	1.5 ± 0.8
LVOT maximal pressure gradient (mmHg)	11 ± 7.9	8.6 ± 3.1	8 ± 2.7
LVOT longitudinal strain (%)	−10.4 ± 3.7	−12.8 ± 1.7	−15.1 ± 2.5

Data are presented as *n* (%) and means ± standard deviation.

## DISCUSSION

This study assessed the feasibility of using 4D-flow CMR in the examination of postoperative haemodynamic differences among HCM patients. As 4D-flow CMR demonstrated consistent results across separate groups, it seems to be a feasible technique for comparing haemodynamic outcomes of the involved different surgical approaches. Regarding the differences between septal myectomy with and without AMVLE, findings can be summarized as follows: patients who underwent myectomy with concomitant AMVLE exhibited (i) lower aortic WSS, (ii) lower LVOT peak velocity, (iii) higher KE in the LVOT and (iv) lower KE in the ascending aorta. Consequently, the myectomy + AMVLE group showed a better trend towards improved haemodynamics closer to the healthy haemodynamic reference values.

These findings are in line with our hypothesis and may be ascribed to the possibly increased openness of the LVOT achieved through additional AMVLE. While the recent American College of Cardiology guidelines [3] do not recommend concomitant MVR during myectomy (class III) because of an elevated risk of adverse events [24], these results may favour the opposite perspective, aligning with studies demonstrating that myectomy with concomitant MVR may yield favourable clinical outcomes [7, 8]. However, it is essential to note that this feasibility study primarily focused on the possibility of 4D-flow CMR to assess differences between these two techniques, rather than on reporting differences that could have potential clinical implications.

Since the use of 4D-flow CMR to assess postoperative haemodynamics in HCM patients remains relatively unexplored, it is essential to examine the clinical validity of its parameters in the future. 4D-flow CMR is extensively employed in clinical research to enhance our comprehension of the impact of cardiovascular diseases on blood flow [25]. Nevertheless, the analysis of 4D-flow CMR data remains complex, and the development of accurate analysis tools that offer consistent quantification of 4D-flow values is still in progress for broader clinical adoption [26]. Nonetheless, our study demonstrates the feasibility of utilizing 4D-flow CMR to compare surgical treatment strategies within a specific patient population and evaluate haemodynamic surgical outcomes. In future studies with larger sample sizes, 4D-flow CMR could emerge as a potential key feature for distinguishing the superiority of different surgical approaches across various cardiac disease domains.

The primary limitation of our pilot study lies in its small sample size and its single-centre nature, making it impossible to carry out statistical analyses and draw generalizable conclusions for potential clinical implications. Also, the case–cohort design in this study might have introduced selection bias since selecting patients with favourable haemodynamic profiles could skew results. In an ideal scenario, we would have assessed the preoperative haemodynamic conditions in these patients to enable the evaluation of haemodynamic changes within the same patient both preoperatively and postoperatively; however, this was not possible since the patients were already operated upon inclusion in this study. Nevertheless, given that this study was intended as a feasibility study, these limitations do not detract from its purpose.

Furthermore, patients in this study who underwent myectomy with concomitant AMVLE had a significantly longer postoperative follow-up duration at the time of their CMR evaluation, compared to the isolated myectomy group. This discrepancy in postoperative follow-up period may have influenced the outcomes in favour of the myectomy + AMVLE group, given that the LV reversed remodelling process, aimed at normalizing LV dimensions, could have potentially progressed with an extended follow-up period after surgery. Nevertheless, literature shows that the LV reversed remodelling process in HCM patients is generally completed within a range of 6–18 months after surgery [27, 28]. Therefore, it is reasonable to assert that the mean follow-up time for both groups falls within or beyond this recognized range. On the other hand, the myectomy + AMVLE group was older compared to the isolated myectomy patients, which could have favoured the isolated myectomy patients on their side, as ageing is associated with an impaired reversed remodelling process [29]. Another interesting observation between the two groups is the notably larger difference in LV myocardial mass between the isolated myectomy and the myectomy + AMVLE groups (192.4 ± 99 g vs. 122.7 ± 14 g, respectively). While this disparity could be attributed to differences in perioperative resected mass, this explanation seems unlikely, as the mean resected mass in the isolated myectomy group is actually higher (7.8 g) than in the myectomy + AMVLE group (6.7 g) (Supplementary Material, Table S1). Therefore, it is more plausible that the observed difference in LV myocardial mass is related to the longer average period of reverse remodelling in the myectomy + AMVLE group.

In conclusion, this study evaluated the feasibility of employing 4D-flow CMR to compare postoperative haemodynamic disparities between obstructive HCM patients undergoing isolated myectomy and those undergoing myectomy with concurrent AMVLE. The findings revealed that 4D-flow CMR is a feasible imaging technique to evaluate haemodynamic differences in this small cohort of HCM patients, who had two different surgical approaches. Although the observed differences in AMVLE patients were superior compared to the isolated myectomy patients, the validity and added value of this imaging technique need to be corroborated in larger studies.

## Supplementary Material

ivae210_Supplementary_Data

## Data Availability

The data that support the findings of this study are available from the corresponding author, S.M., upon reasonable request. Due to privacy or ethical restrictions, access to some data may be limited.

## ABBREVIATIONS

3D: Three-dimensional
4D: four-dimensional
AMVLE: Anterior mitral valve leaflet extension
AV: Aortic valve
CMR: Cardiac magnetic resonance imaging
HCM: Hypertrophic cardiomyopathy
KE: Kinetic energy
LV: Left ventricle
LVOT: Left-ventricular outflow tract
MV: Mitral valve
MVR: Mitral valve repair
SSFP: Steady-state free precession
WSS: Wall shear stress
