# Supplementary material for: Feasibility of 4D-flow CMR for haemodynamic characterization in hypertrophic cardiomyopathy after septal myectomy with and without anterior mitral valve leaflet extension
Source: Interdiscip Cardiovasc Thorac Surg. 2024 Dec 16;40(1):ivae210. doi: 10.1093/icvts/ivae210 (PMC11852344; doi:10.1093/icvts/ivae210)
Supplement: ivae210_Supplementary_Data [file ivae210_supplementary_data.docx]

| Preoperative patient details | | Gender | Age *(years)* | HCM  phenotype | Maximum septal thickness *(mm)* | LVOT peak gradient in rest *(mmHg)* | LVOT peak gradient in exercise *(mmHg)* | Clinical  symptoms | Surgical resected mass *(grams)* | Postoperative follow-up *(years)* |
| --- | --- | --- | --- | --- | --- | --- | --- | --- | --- | --- |
| Isolated myectomy | |  |  |  |  |  |  |  |  |  |
|  | Patient 1 | Female | 58 | Basal | 16 | 34 | 75 | Dyspnea d’effort | 6 | 1 |
|  | Patient 2 | Male | 50 | Basal to apical | 32 | 19 | 122 | Dyspnea & dizzy at exercise | 9 | 0.7 |
|  | Patient 3 | Female | 69 | Basal | 18 | 107 | 118 | Dyspnea d’effort | 8.5 | 0.5 |
| Myectomy + AMVLE | |  |  |  |  |  |  |  |  |  |
|  | Patient 4 | Male | 65 | Basal | 22 | 100 | *NA* | Dyspnea &  near-collaps | 9 | 3.9 |
|  | Patient 5 | Female | 62 | Basal | 16 | 17 | 88 | Dyspnea d’effort | 7 | 2.2 |
|  | Patient 6 | Female | 73 | Basal | 16 | 24 | 79 | Dyspnea &  near-collaps | 4 | 2.1 |
| Data are presented as n (%) and means ± standard deviation  *Abbreviations: HCM; hypertrophic cardiomyopathy, LVOT; left ventricular outflow tract* | | | | | | | | | | |
